# Treatment paths for localised prostate cancer in Italy: The results of a multidisciplinary, observational, prospective study (Pros-IT CNR)

**DOI:** 10.1371/journal.pone.0224151

**Published:** 2019-11-01

**Authors:** Michela Buglione, Marianna Noale, Alessio Bruni, Alessandro Antonelli, Filippo Bertoni, Renzo Corvo’, Umberto Ricardi, Paolo Borghetti, Marta Maddalo, Claudio Simeone, Ercole Mazzeo, Angelo Porreca, Sergio Serni, Pierfrancesco Bassi, Mauro Gacci, Vincenzo Mirone, Rodolfo Montironi, Andrea Tubaro, Alfredo Berruti, Giario Natale Conti, Stefania Maggi, Stefano Maria Magrini, Luca Triggiani, the Pros-IT CNR study group

**Affiliations:** 1 Radiation Oncology Department, University and Spedali Civili Hospital, Brescia, Italy; 2 National Research Council, Neuroscience Institute, Aging Branch, Padua, Italy; 3 Radiotherapy Unit, Department of Oncology and Hematology, University Hospital of Modena, Modena, Italy; 4 Department of Urology, University and Spedali Civili Hospital, Brescia, Italy; 5 Department of Radiation Oncology, University Hospital “Policlinico San Martino”, Genoa, Italy; 6 Radiation Oncology Unit, Department of Oncology, School of Medicine—University of Turin, Turin, Italy; 7 Department of Urology, University and Spedali Civili Hospital, Brescia, Italy; 8 Department of Robotic Urological Surgery, Abano Terme Hospital, Abano Terme, Padua, Italy; 9 Department of Urologic Robotic Surgery and Renal Transplantation, Careggi University Hospital, Florence, Italy; 10 Department of Urology, Catholic University of Rome, Policlinico Gemelli, Rome, Italy; 11 Urology Unit, University Federico II, Naples, Italy; 12 Section of Pathological Anatomy, Polytechnic University of the Marche Region, School of Medicine–Ospedali Riuniti, Ancona, Italy; 13 Urology Unit, Sant'Andrea Hospital, "La Sapienza" University, Rome, Italy; 14 Medical Oncology Unit, ASST-Spedali Civili, Department of Medical and Surgical Specialties, Radiological Sciences, and Public Health, University of Brescia, Brescia, Italy; 15 Urology Unit, ASST Lariana, Sant'Anna Hospital, Como, Italy; The Cancer Institute of New Jersey, Robert Wood Johnson Medical School, UNITED STATES

## Abstract

**Background:**

There are several treatments available to newly diagnosed prostate cancer (PCA) patients. Although surgery and radiotherapy (RT) with or without androgen deprivation therapy (ADT) are widely adopted treatment options for localized PCA together with active surveillance (AS), there is no consensus nor randomised trials on treatment selection, prospective quality of life (QOL), along with toxicity outcomes and according to treatment modality in the Italian population. The current study aimed to describe clinical-therapeutic features and QOL at PCA diagnosis, according to different treatment patterns in a large prospective, Italian population, enrolled in the Pros-IT CNR study.

**Methods:**

The Pros-IT CNR is an on-going national, multicenter, observational, prospective study on patients affected by PCA who have been referred by 97 Italian Urology, Radiation Oncology and Medical Oncology facilities participating in the project. The possible relationships between the treatment patterns reported in the 6 month follow-up case report form and patients’ features at diagnosis were evaluated using exploratory multiple correspondence analysis (MCA) and other data analysis method.

**Results:**

At diagnosis, surgery and AS patients were significantly younger, had fewer comorbidities, lower PSA levels and Gleason Score (GS) values; they were also diagnosed at an earlier stage of disease with respect to the RT or ADT patients who showed significantly worse QoL scores at the time of diagnosis.

**Conclusions:**

An analysis of the data collected at baseline and 6 months later uncovered substantial differences in ages, comorbidities, clinical and QOL features in the various treatment groups. These findings do not fully reflect the current PCA treatment guidelines and suggest the need for a multidisciplinary consensus guideline to ameliorate both the counselling and treatments of PCA patients.

## Introduction

Even if national and international guidelines consider both surgery and radiotherapy (RT) as treatments of choice for localized Prostate Cancer (PCA) [1–5], no randomized trials compared their efficacy in different risk groups, with the exception of Hamdy’s study which examined 10-year outcomes of localized PCA treatments [6]. Active surveillance (AS), with the advantage of avoiding radical treatments side effects is also considered an option for patients affected by low/very low risk PCA [7–8].

Patients choosing to be treated may ultimately decide for themselves which treatment to undertake only after having received an adequate counselling and after having shared their decision with doctors [9]. Indeed, they may be confused by differing opinions experiencing the ‘lost patient syndrome’ that may get even worse if there is a lack of communication between different members of a multidisciplinary team (MDT) [10], even if managing patients with PCA in a MDT is considered desirable [11].

The study aims to assess the association of clinical and quality of life (QOL) characteristics with different patterns of care in a sample of PCA patients in Italy.

## Materials and methods

### “Pros-IT CNR” study

The design of the Pros-IT CNR study has been described elsewhere [12–13]. Briefly, this on-going national, multicenter, observational, prospective, study was designed to monitor QoL in a sample of treatment-naïve Italian patients with PCA diagnosed between 2014 and 2015. Ninety-seven centers (urology, radiation and medical oncology facilities) located in Italy, enrolled 1705 consecutive patients: 949 in urology, 717 in radiation oncology and 39 in medical oncology departments. A baseline evaluation at the time PCA was diagnosed (and the patient was enrolled), and, 6, 12, 24, 36, 48 and 60 months later were/are foreseen for protocol [14]. Complete information about the chosen treatment was available at the 6 months follow up for the vast majority of patients enrolled (1493 patients at 6 months follow up, 97% of expected).

### Ethics

The study protocol was approved by the Ethics Committee of the clinical coordinating center (Sant’Anna Hospital, Como, Italy; register number 45/2014) and by those of the other participating centers. The study was carried out in accordance with the principles of the declaration of Helsinki. All the participants signed an informed consent form.

### Study population

**Fig 1:**
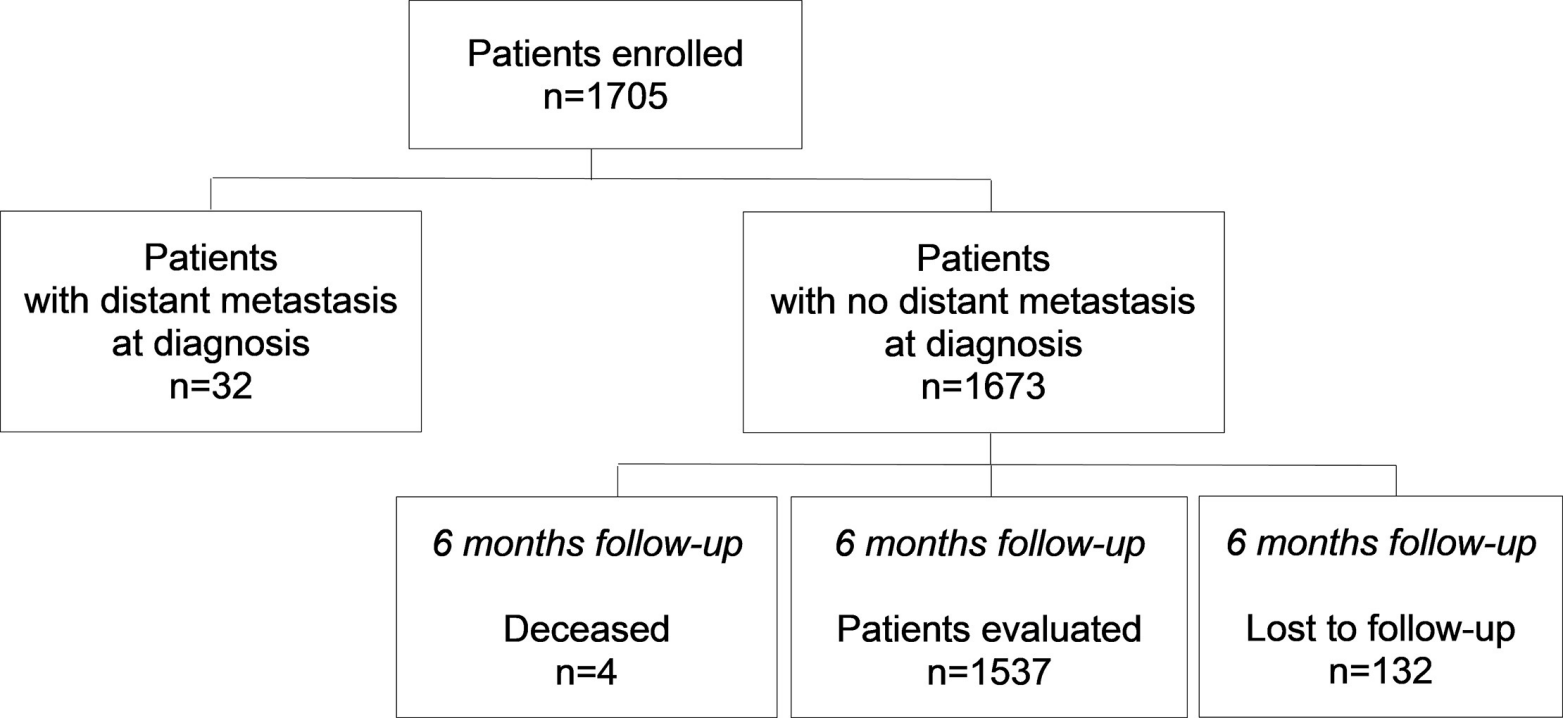
Patients enrolled in the Pros-IT CNR study from prostate cancer diagnosis to the 6 months follow-up.

In the Pros-IT CNR study, 1705 patients were enrolled. For the present analysis 6-month follow-up data were available for 1537 patients without distant metastasis (while 32 had distant metastasis at diagnosis, 4 died and 132 were lost to follow-up) (Fig 1).

### Data collection

Demographics/anamnestic data (weight, height, smoking status), comorbidities evaluated using the Cumulative Illness Rating Scale (CIRS) [15], pharmacological treatments, initial diagnosis, tumor stage and QOL scores were evaluated at the baseline (time of diagnosis). Information on life status, treatments prescribed and QOL are/were collected during follow-up evaluations. Characteristics of institution where the prostate cancer was diagnosed (presence of Urology unit, Radiation Oncology Unit, Medical Oncology Unit and/or Prostate Cancer Unit) as well as physician who enrolled the patient in the study (urologist,radiation oncologist or medical oncologist) were also collected.

### Outcome measures analyzed

Patients’ QOL was assessed at diagnosis and then during each follow-up evaluation using the Italian version of the University of California Los Angeles-Prostate Cancer Index [16], which measures health-related QOL in PCA by investigating six domains: urinary function and bother (UF, UB), bowel function and bother (BF, BB), sexual function and bother (SF, SB). Responses were scored from 0 to 100, and higher score mirror better QOL.

Additionally, the Italian version of the Short-Form Health Survey (SF-12 Standard v1 scale) [17] was administered. SF-12 includes physical/mental component subscales (PCS and MCS, respectively) both ranging from 0 to 100, with higher scores indicating better self-perceived health states.

### Statistical analysis

Data were analyzed without imputation of missing values. Categorical variables were presented as numbers and percentages. Continuous variables were reported as means and standard deviations (SD) or medians and quartile 1 (Q1) and quartile 3 (Q3). Normal distributions for continuous variables were tested using the Shapiro-Wilk test.

The patients’ features at diagnosis, as well as the characteristics of institution where the prostate cancer was diagnosed (presence of Urology, Radiation Oncology or Medical Oncology Unit and/or Prostate Cancer Unit) were compared according to the different treatments selected for PCA applying Fisher's exact test or Chi-squared test for categorical variables; the Wilcoxon rank-sum test was employed to analyze the continuous variables. Post-hoc analyses with Bonferroni adjustment for multiple comparison were applied.

Exploratory multiple correspondence analysis (MCA) was performed to evaluate the relationships among patients ‘characteristics at diagnosis to identify specific profiles [18–19]. MCA permits viewing graphically the relationships among variables, by defining a map of cross-tabulations where rows and columns are represented as profiles in multidimensional space. In MCA, active variables were used to search for the factorial solution (inertia), and included age, education, marital status, smoking status, family history of PCA, presence of diabetes, comorbidities, T stage, Gleason Score (GS), PSA level and characteristics of institution where the prostate cancer was diagnosed (presence of Urology, Radiation Oncology, Medical Oncology Unit, Prostate Cancer Unit). PCA treatments prescribed during the 6 months following diagnosis were considered as supplementary variables. A p-value of less than 0.05 for a 2-sided test was considered statistical significant. All the analyses were performed using SAS 9.4 software. Full data were presented at the Uro-Oncological Study Group Meeting during the Italian Radiation Oncologist National Conference recently held in Rimini (27–29 September 2019).

## Results

**Fig 2:**
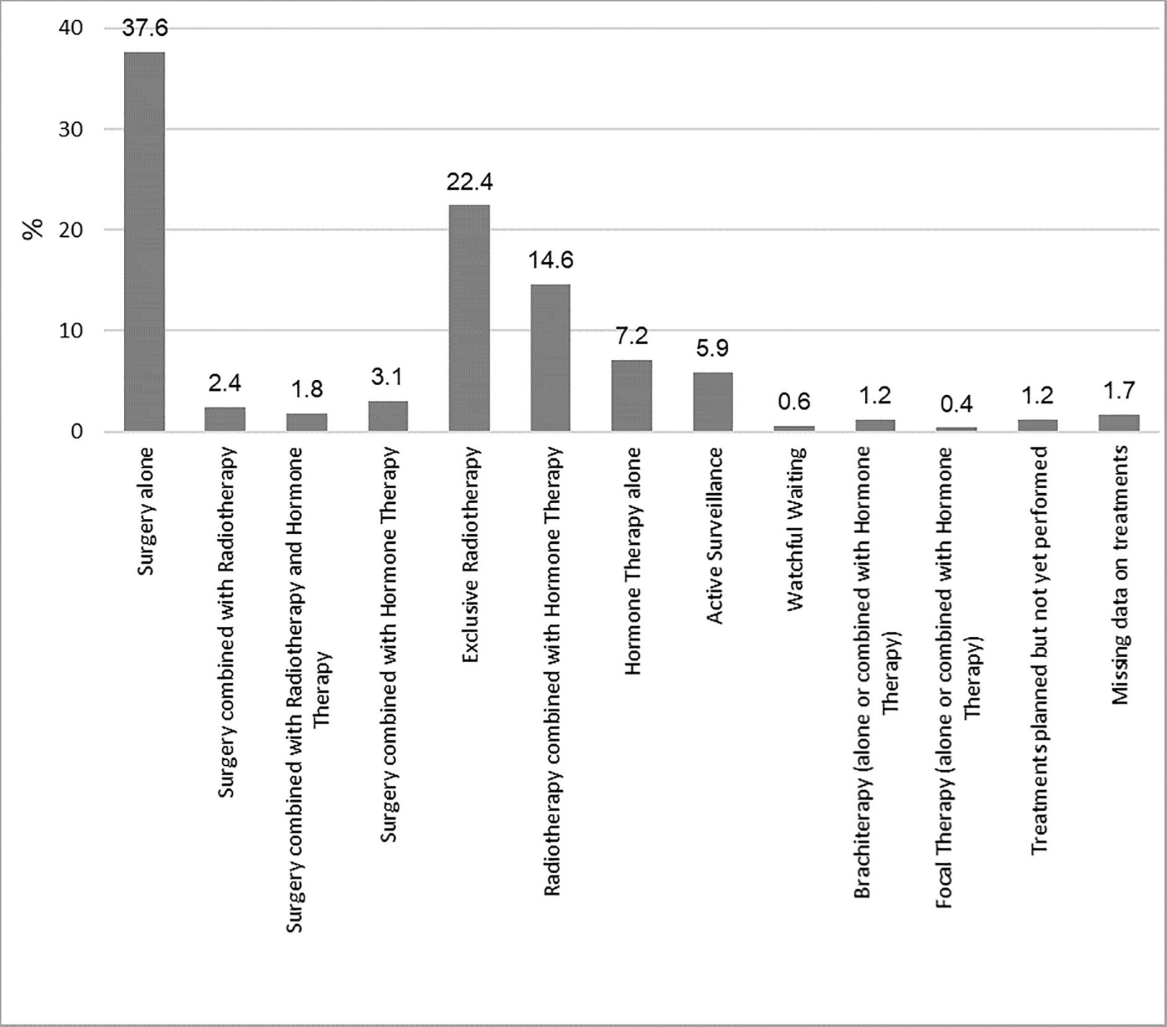
Prostate cancer treatments reported at the follow-up at 6 months.

Treatment was stratified as follows: surgery alone (37.6%); surgery and RT (2.4%); surgery plus RT plus Androgen Deprivation Therapy (ADT; 1.8%); surgery and ADT (3%); exclusive RT or RT plus ADT (22% and 15%, respectively), ADT alone (7%), AS (6%) and brachytherapy (BT) (1%) (Fig 2). Information on treatments carried out during the six-month period following diagnosis was unavailable for 26 patients. The treatment groups included in these descriptive analyses were: surgery alone, surgery combined with RT, surgery combined with RT and ADT, exclusive RT, RT combined with ADT, AS and ADT alone; a total sample size of 1412 patients was considered for the present study.

### Treatment features

Overall, 690 patients without distant metastasis at diagnosis underwent surgery for PCA. Of these, 58.8% underwent a robot-assisted radical prostatectomy (RARP), 24.1% an open radical prostatectomy (ORP), 15.7% a laparoscopic prostatectomy and 0.4% a palliative transurethral resection of the prostate. Information on the surgical approach was unavailable for less than 1% of patients. Three hundred seventy-six patients (57.6%) underwent nerve-sparing surgery and 328 (47.5%) lymphadenectomy.

RT with external beams was delivered to 634 patients of whom 75% underwent Image-Guided Radiation Therapy (IGRT). A three-dimensional conformal radiotherapy (3D-CRT) technique was used in 200 patients (33.2%), Intensity-Modulated Radiation Therapy (IMRT) in 248 (41.2%), Volumetric Modulated Arc Therapy (VMAT) in 147 (24.4%) and Stereotactic body radiotherapy (SBRT) in 7 (1.2%). The volume treated included prostate alone in 142 patients (24.7%), prostate plus seminal vesicles in 340 patients (59.2%) and prostate, seminal vesicles and pelvic nodes in 92 (16%). Active Surveillance was adopted in 90 patients (5.9%), while 413 received ADT: LH-RH agonist (215; 56.1%) was the most frequently prescribed ADT. LH-RH antagonists were prescribed to 55 patients; peripheral antiandrogen drugs to 43 and total androgenic blockade to 70.

### Correlations between patient’s characteristics and therapeutic features

Patients’ characteristics at diagnosis, stratified by treatments received until follow-up at 6 months are outlined in S1 Table. Patients undergoing surgery alone or combined with RT or with RT/ADT were the youngest, along with those candidates to AS, followed by patients on RT and those on ADT (p<0.0001). The prevalence of diabetes at diagnosis was 28.4% among patients on ADT alone, 22.8% among those on RT combined with ADT, 17.5% among those on exclusive RT and was ≤ 10% among those on surgery alone or AS (p<0.0001 across groups). Higher percentages of patients undergoing ADT alone, RT combined with ADT or exclusive RT had three or more moderate/severe comorbidities according to CIRS (22.2%, 19.7% and 18%, respectively), compared to patients on AS or surgery (14.6% and 10.7%, respectively). They also reported taking a higher median number of drugs taken (2, 3 and 2), compared to patients undergoing surgery alone or AS (1 and 1; p = 0.0013 and p<0.0001, respectively). There were no significant differences in the groups in the obesity prevalence rates.

The median PSA value at diagnosis was 6.5 ng/mL (Q1 = 5, Q3 = 9.1) and 6.2 ng/mL (Q1 = 4.8, Q3 = 7.7) in the surgery alone and AS groups, respectively. These values were significantly lower than those in the RT alone (7.0 ng/mL, Q1 = 5.2, Q3 = 10) and ADT (10.2 ng/mL, Q1 = 7, Q3 = 21) groups. Ninety-three percent of patients in AS, 50.8% in surgery alone, 45.9% in RT alone, and 17.8% in ADT alone groups had a GS ≤ 6 at diagnosis (p<0.0001).

Considering the physician who enrolled each patient, 94.8% of patients in the surgery alone group were enrolled by urologists, 4.8% by radiation oncologists and 0.4% by medical oncologists. Conversely, 89.9% of patients in the RT group were enrolled by radiation oncologists, 8.4% by urologists and 1.7% by medical oncologists. Patients in the RT combined with ADT group were enrolled by urologists in 47.3% of cases, by radiation oncologists in 44.6% of cases and by medical oncologists in 8.2% of cases.

As far as QOL at diagnosis was concerned, the patients in surgery alone or AS groups had better UF, BF, SF, SF12 PCS scores than the others (p<0.05). As described in Porreca A et al.[13], features at diagnosis associated with lower SF12 PCS scores (i.e. worst physical component scores) were older age, obesity, the presence of three or more moderate/severe comorbidities, having a Gleason score at diagnosis of ≥8, living in Southern regions of Italy and being widowed or single. Patients’ characteristics at diagnosis associated with lower SF12 MCS scores (i.e. worst mental component score) were younger age, the presence of three or more moderate/severe comorbidities and having a T-score at diagnosis higher than T1. The main characteristic associated with lower UCLA-PCI scores was older age; furthermore, lower sexual function scores were associated with the presence of diabetes, three or more moderate/severe comorbidities, a T-score at diagnosis higher than T1 or a Gleason score of >8.

**Fig 3:**
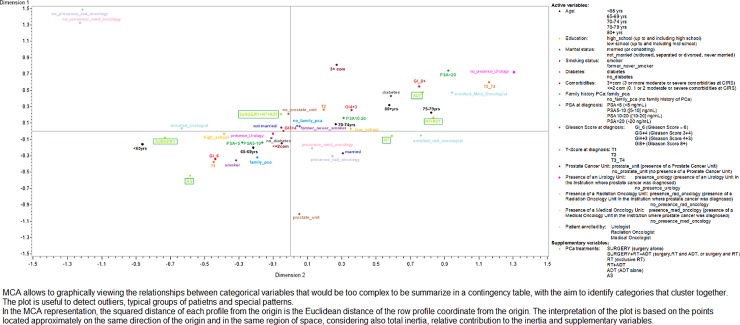
Multiple Correspondence Analysis of PCA patients’ characteristics at diagnosis.

MCA analysis showed that inertia was decomposed along two principal dimensions (Fig 3). The first axis accounted for 53% of the inertia and the second for a further 15%, giving a cumulative inertia of 68%. Ten modalities contributed to almost 60% of the variance of the axis 1 (*dimension 1)*: on the right of the axis, the most important modalities were PSA ≥ 20 ng/mL at diagnosis, GS ≥ 8, T3 or T4 staging, being 75–79 at diagnosis, having diabetes and having been enrolled by a medical or a radiation oncologist or in an Institution with no presence of an Urology Unit; to the left side, the most important modalities were T1 staging, GS ≤ 6, and being under 65 years at diagnosis. Axis 1 grouped together the diagnosis severity (GS, T staging) with age and diabetes. Four modalities contributed to 60% of the variance of the axis 2 (*dimension 2)*: having 3 or more comorbidities at diagnosis, having being diagnosed in an Institution without Radiation or Medical Oncology Unit were towards the top of the axis, while the main contributors towards the bottom was having been diagnosed in an Institution with a Prostate Cancer Unit. Supplementary variables (in this case, the treatment strategy selected) did not contribute to determining the solution, but they were projected onto the axis to facilitate interpretation of the analytical solution. AS and surgery alone were close to one other on the third quadrant, together with a GS ≤ 6, T1 staging, having at most two comorbidities, no diabetes, being younger than 69 years at the time of diagnosis and having being diagnosed in an Institution with an urology unit. RT was plotted on the fourth quadrant, together with the presence of a Medical or a Radiation Oncology Unit. ADT alone and RT plus ADT were plotted on the first quadrant, near to ≥ 80 years at diagnosis, a GS ≥ 8, T3/T4, PSA ≥20 ng/ml and having been enrolled by a medical oncologist. Surgery combined with RT and ADT was plotted on the second quadrant, far enough from the other factors, but near to the lack of a Prostate Cancer Unit in the Institution that performed the initial diagnosis. We chose not to consider QOL-related factors as active variables, because they were self-reported and their inclusion in the analysis did not substantially increase the inertia explained by the first two dimensions (70% vs 68%).

## Discussion

The Pros-IT CNR study is mainly devoted to analyze the impact of patients’ and treatments related features on QOL, and future analyses will evaluate changes in QOL following different prostate cancer treatments. The present work aimed to analyze data collected, considering patients’ clinical features at diagnosis, according to the treatment path. The study structure has been conceived to take a picture of the “real world scenario” of Italian PCA patients treated in different centers at time of diagnosis, allowing also to consider the clinical behavior of Italian urologists and oncologists when choosing treatment for individual patient. The current study uncovered that surgery alone closely followed by RT alone were the more frequently given treatments, whereas ADT alone and AS were less frequently adopted. This finding is not unexpected as some authors have reported that surgical treatments have long been the most frequently adopted strategy in Italy for PCA [20]. These findings are in line with a recent US study, based on the Surveillance, Epidemiology and End Results (SEER) database, showing that radical prostatectomy is the most commonly performed procedure (37% of patients) [21]. In the present series, surgery was found to be the preferred treatment for the youngest patients without significant comorbidities. Indeed, patients undergoing surgery alone were younger than those prescribed exclusive RT and ADT alone. Of the 690 patients who underwent radical prostatectomy, 58.8% underwent RARP, 24.1% ORP and 15.7% laparoscopic procedure. The pattern may also be linked to some studies reporting that RARP can reduce post-operative morbidity, hospital re-admissions and total admission time and can thus affect the QoL of these patients [22–24]. Coughlin et al [24] recently conducted a phase III randomized trial analyzing functional and oncological postoperative outcomes up to 24 months after RARP or ORP. There were no statistically significant differences in sexual functions, urinary distress or clinical outcome, although the former showed a slightly better biochemical relapse free survival. The Authors concluded that both RARP and ORP yielded similar functional outcomes at 24 months, but they advised using caution in interpreting the oncological outcomes [25]. Pros-IT CNR data also support this conclusion [26].

In our data, 2.4% of patients underwent surgery followed by adjuvant RT, 1.8% surgery, RT and ADT, 3% surgery and ADT, the latter in particular when the patient was characterized by high risk features (T3 and GS >7).

Studies comparing cost-effectiveness and QOL in patients undergoing surgery *vs* RT *vs* combined modality treatments have produced conflicting findings. While one report published in 2012 favored RT plus ADT for high-risk PCA [27], another one published in 2013 supported the use of surgery for intermediate-to-high risk PCA [28]. When Dorth et al. conducted a cost effectiveness analysis on intermediate-to-high risk PCA and compared RT plus ADT *vs* surgery using a Markov model, their data showed that the former was characterized by better results in terms of clinical outcome and quality-adjusted life expectancy [29–30]. An increase in the fraction of high-risk patients treated with surgery has been reported also by the aalysis of the SEER database for the years 2010–2015; this increase does not seem completely justified by the current guidelines [31].

In Italy, an increase in the use of External Beam RT (EBRT) for curative treatment of PCA has been documented in two subsequent patterns of practice studies run under the aegis of Italian Association for Radiotherapy and Clinical Oncology (AIRO) [32–33]. In the present study, patients treated with EBRT have been referred to radiation oncologist for the most by urologists. Seventy-five percent underwent IGRT; 33.2% 3D-CRT technique, 41.2% IMRT, 24.4% VMAT, 1.2% SBRT. These results seem to confirm that the new technologies are increasingly being used by Italian Radiation Oncology Centers. In comparison with previous patterns of practice studies supported by AIRO, the percentage of PCA patients treated with new RT technologies has increased considerably over recent years, confirming the conclusion that IGRT can improve RT treatment accuracy [34] and may reduce severe acute and late urinary/rectal side effects [35]. Furthermore, dose escalation, which IMRT/VMAT techniques have greatly facilitated, has become increasingly commonplace in PCA patients in randomized trials demonstrating improved disease control when increasing RT dose are applied [36–37]. More recent randomized trials have reported that moderately hypofractionated regimens are equally effective and less disagreeable to patients compared to standard regimens since they reduce total treatment time. These finding seem to support the use of more sophisticated RT technologies (IMRT/VMAT) and IGRT techniques [38–40]. Also, in Italy, an increased use of moderately hypofractionated, IGRT treatments has been observed in recent years (Table 1). Finally, although extreme hypofractionated SBRT has been attracting growing interest, convincing but less robust evidence has not led to a larger use [40–41].

**Table 1:** Evolution of techniques and doses 1985 through 2015, according to different subsequent multicentric databases.

Technique/Dose	Patterns of practice I (1985–98)[31] 1759 patients	Patterns of practice II (1999–2003)[32] 3001 patients	Patterns of practice III (2004–2011) 2300 patients*	Pros- IT CNR (2014–2015) 634 patients^§^
2D, n (%)	1315 (75)	725 (24)	-	-
3D, n (%)	444 (25)	2269 (75)	1905 (83)	200 (33)
IMRT, n (%)	-	7 (1)	395 (17)	248 (42)
VMAT, n (%)	-		Not evaluated	147 (24)
SBRT, n (%)	-		-	7 (1)
Dose Gy, n (%)				
< 70	728 (42)	218 (7)	24 (1)	107 (20)
70–75	1021 (58)	2531 (84)	1255 (70)	234 (46)
≥ 76	-	252 (9)	729 (29)	174 (34)
IGRT	-	-	334 (15)	476 (75)

*data presented at Urological study group meeting during AIRO Conference 2019

^§^present paper

In the United States (US) a progressive decline in BT was registered between 2004 and 2014 [21]. BT is available in some centers. However, the number of patients participating in the Pros-IT CNR study who were prescribed this therapy was very small (1%) and reflects its scarce diffusion at this moment in Italy. A recently published article, based on data from a large multicenter Italian database, has confirmed the safety and efficacy of prostate BT [42]. Despite the many advantages of BT (shorter duration, good results and cost effectiveness), the explanation for its limited diffusion may be found in the scarcity of adequately trained radiation oncologists also due to a relatively long learning curve [43].

In the recent years, a concomitant increase in the choice not to give definitive treatment was also noted [21]. In the US, very recent data on a population of 164.760 PCA patients from SEER database show an increase from 8.1% to 15.8% in AS as initial management from 2010 through 2015; this management strategy was applied to 42.1% of low risk patients in 2015 [31]. In our study, AS was adopted in 6% of the cases: according to the main guidelines [1–5], a lower than expected fraction of patients with early stage PCA has been addressed to AS and BT. At the same time, it seems that an excess of advanced stage patients has firstly been surgically treated as well as an excess of radical treatments for low/very low risk patients has therefore been proposed.

Several limitations of our study should be acknowledged: participating centers were involved on a voluntary basis and, therefore, a selection bias cannot be excluded; information on factors that could also influence physicians’ or patients’ treatment choice, including Magnetic Resonance Imaging, objective assessment of preoperative voiding status, volume of the prostate, patients’ access to health care services, and surgeon’s experience, were not available in the Pros-IT CNR study.

Concluding, the Pros-IT CNR study can be considered a remarkable forum for Italian urology and oncology specialists to analyze and discuss the latest trends and patterns of care for PCA. Future studies will help to clarify the efficacy of treatment strategies in different risk groups and the baseline criteria to be used to select the most appropriate treatment path for each patient.

## Supporting information

S1 Table Patients’ characteristics stratified according to the treatment pathway. (DOC)

## References

[1] NCCN. 2018 NCCN Clinical practice guidelines in oncology, Prostate Cancer, Version 4.2018. nccn.org2017, August 15, 2018.

[2] EAU. EAU Guidelines. Edn. presented at the EAU Annual Congress Copenhagen 2018 EAU Guidelines Office: Arnhem, The Netherlands, 2018.

[3] Parker C, Gillessen S, Heidenreich A, Horwich A, on behalf of the ESMO Guidelines Committee ESMO. Cancer of the prostate: ESMO Clinical Practice Guidelines for diagnosis, treatment and follow-up. Annals of Oncology 2015; 26 (Supplement 5): v69–v77. 26205393 10.1093/annonc/mdv222

[4] AIRO. Linee guida carcinoma della prostata-AIRO 2016, Tumori 2016; Special Issue 1: S1–S79.

[5] AIOM. 2018, Linee guida carcinoma della prostata. Available at: https://www.aiom.it/wp-content/uploads/2018/11/2018_LG_AIOM_Prostata.pdf.

[6] Hamdy FC, Donovan JL, Lane JA, Mason M, Metcalfe C, Holding P et al, ProtecT Study Group. 10-Year Outcomes after Monitoring, Surgery, or Radiotherapy for Localized Prostate Cancer. *N Engl J Med* 2016; 375: 1415–1424. 10.1056/NEJMoa1606220 27626136

[7] Briganti A, Fossati N, Catto JWF, Cornford P, Montorsi F, Mottet N et al Active Surveillance for Low-risk Prostate Cancer: The European Association of Urology Position in 2018. Eur Urol 2018; 74: 357–368. 10.1016/j.eururo.2018.06.008 29937198

[8] Chen RC, Rumble RB, Loblaw DA, Finelli A, Ehdaie B, Cooperberg MR et al Active Surveillance for the Management of Localized Prostate Cancer (Cancer Care Ontario Guideline): American Society of Clinical Oncology Clinical Practice Guideline Endorsement. J Clin Oncol 2016; 34: 2182–2190. 10.1200/JCO.2015.65.7759 26884580

[9] Mohler J, Bahnson RR, Boston B, Busby JE, D'Amico A, Eastham JA et al. NCCN clinical practice guidelines in oncology: prostate cancer. *J Natl Compr Canc Netw* 2010; 8:162–200. 10.6004/jnccn.2010.0012 20141676

[10] Denis LJ, Roobol M, Dourcy-Belle Rose B. Prostate cancer for the horizon of the patient. *Acta Oncol* 2011; 50 (Suppl. 1): 148–154. 10.3109/0284186X.2010.528446 21604956

[11] Valdagni R, Albers P, Bangma C, Drudge-Coates L, Magnani T, Moynihan C et al. The requirements of a specialist Prostate Cancer Unit: a discussion paper from the European School of Oncology. *Eur J Cancer* 2011; 47:1–7. 10.1016/j.ejca.2010.10.029 21126868

[12] Noale M, Maggi S, Artibani W, Bassi PF, Bertoni F, Bracarda S et al Pros-IT CNR: an Italian prostate cancer monitoring project. *Aging Clin Exp Res* 2017; 29: 165–172. 10.1007/s40520-017-0735-6 28236267

[13] Porreca A, Noale M, Artibani W, Bassi PF, Bertoni F, Bracarda S et al Disease specific and general health-related quality of life in newly diagnosed prostate cancer patients: the Pros-IT CNR study. *Health Qual Life Outcomes* 2018; 16:122 10.1186/s12955-018-0952-5 29898750 PMC6001046

[14] Gacci M, Noale M, Artibani W, Bassi PF, Bertoni F, Bracarda S et al Quality of Life After Prostate Cancer Diagnosis: Data from the Pros-IT CNR.*Eur Urol Focus* 2017; 3:321–324. 10.1016/j.euf.2017.10.009 29146557

[15] Conwell Y, Forbes NT, Cox C, Caine ED. Validation of a measure of physical illness burden at autopsy: the Cumulative Illness Rating Scale. *J Am Geriatr Soc* 1993; 41: 38–41. 10.1111/j.1532-5415.1993.tb05945.x 8418120

[16] Gacci M, Noale M, Artibani W, Bassi PF, Bertoni F, Bracarda S et al Quality of life after radical treatment of prostate cancer: validation of the Italian version of the University of California Los Angeles-Prostate Cancer Index. *Urology* 2005; 66: 338–343. 10.1016/j.urology.2005.02.027 16098363

[17] Apolone G, Mosconi P, Quattrociocchi L, Gianicolo EAL, Groth N, Ware J JE. Questionario sullo stato di salute SF-12 Versione Italiana. Milano, Guerini e Associati Editore, 2001.

[18] Greenacre MJ. *Theory and Applications of Correspondence Analysis*. UK Academic Press, London, 1984.

[19] Sourial N1, Wolfson C, Zhu B, Quail J, Fletcher J, Karunananthan S et al. Correspondence analysis is a useful tool to uncover the relationships among categorical variables. *J Clin Epidemiol* 2010; 63: 638–646. 10.1016/j.jclinepi.2009.08.008 19896800 PMC3718710

[20] Trama A, Botta L, Nicolai N, Rossi PG, Contiero P, Fusco M et al Prostate Cancer High Resolution Study Working Group. Prostate cancer changes in clinical presentation and treatments in two decades: an Italian population-based study. *Eur J Cancer* 2016; 67: 91–98. 10.1016/j.ejca.2016.07.021 27620947

[21] Burt LM, Shrieve DC, Tward JD. Factors influencing prostate cancer patterns of care: An analysis of treatment variation using the SEER database. *Adv Radiat Oncol* 2018; 3: 170–180. 10.1016/j.adro.2017.12.008 29904742 PMC6000225

[22] Aning JJ, MacKenzie KR, Fabricius M, McColl E, Johnson MI, Tandogdu Z et al. Detailed analysis of patient-reported lower urinary tract symptoms and effect on quality of life after robotic radical prostatectomy. Urol Oncol 2018; 36: 364.e15-364.e22 10.1016/j.urolonc.2018.05.017 29891407

[23] Ficarra V, Novara G, Rosen RC, Artibani W, Carroll PR, Costello A et al Systematic review and meta-analysis of studies reporting urinary continence recovery after robot-assisted radical prostatectomy. Eur Urol 2012; 62:405–417. 10.1016/j.eururo.2012.05.045 22749852

[24] Jaulim A, Srinivasan A, Hori S, Kumar N, Warren AY, Shah NC et al A comparison of operative and margin outcomes from surgeon learning curves in robot assisted radical prostatectomy in a changing referral practice. *Ann R Coll Surg Engl* 2018; 100: 226–229. 10.1308/rcsann.2018.0001 29484935 PMC5930106

[25] Coughlin GD, Yaxley JW, Chambers SK, Occhipinti S, Samaratunga H, Zajdlewicz L et al Robot-assisted laparoscopic prostatectomy versus open radical retropubic prostatectomy: 24-month outcomes from a randomised controlled study. *Lancet Oncol* 2018; 19: 1051–1060. 10.1016/S1470-2045(18)30357-7 30017351

[26] Antonelli A, Palumbo C, Noale M, Porreca A, Maggi S, Simeone C et al Impact of Surgical Approach on Patient-Reported Outcomes after Radical Prostatectomy: A Propensity Score-Weighted Analysis from a Multicenter, Prospective, Observational Study (The Pros-IT CNR Study). Urol Int 2019; 7: 1–11. 10.1159/000496980 30731456

[27] Parikh R, Sher DJ. Primary radiotherapy versus radical prostatectomy for high-risk prostate cancer: a decision analysis. *Cancer* 2012; 118: 258–267. 10.1002/cncr.26272 21720990

[28] Cooperberg MR, Ramakrishna NR, Duff SB, Hughes KE, Sadownik S, Smith JA et al Primary treatments for clinically localized prostate cancer: a comprehensive lifetime cost-utility analysis. *BJU Int* 2013; 111: 437–450. 10.1111/j.1464-410X.2012.11597.x 23279038 PMC3587031

[29] Dorth JA, Lee WR, Chino J, Abouassaly R, Ellis RJ, Myers ER. Cost-Effectiveness of Primary Radiation Therapy Versus Radical Prostatectomy for Intermediate- to High-Risk Prostate Cancer. *Int J Radiat Oncol Biol Phys* 2018; 100: 383–390. 10.1016/j.ijrobp.2017.10.024 29353655

[30] Borghetti P, Spiazzi L, Cozzaglio C, Pedretti S, Caraffini B, Triggiani L et al. Postoperative radiotherapy for prostate cancer: the sooner the better and potential to reduce toxicity even further. *Radiol Med* 2018; 123: 63–70. 10.1007/s11547-017-0807-x 28924967

[31] Mahal BA, Butler S, Franco I, Spratt DE, Rebbeck TR, D'Amico AV et al. Use of Active Surveillance or Watchful Waiting for Low-Risk Prostate Cancer and Management Trends Across Risk Groups in the United States, 2010–2015. *JAMA*, Published online February 11, 2019. 10.1001/jama.2018.19941 PMC6439610 30743264

[32] Magrini SM, Bertoni F, Vavassori V, Villa S, Cagna E, Maranzano E et al Practice patterns for prostate cancer in nine central and northern Italy radiation oncology centers: a survey including 1759 patients treated during two decades (1980–1998). *Int J Radiat Oncol Biol Phys* 2002; 52: 1310–1319. 10.1016/s0360-3016(01)02783-3 11955744

[33] Pegurri L, Buglione M, Girelli G, Guarnieri A, Meattini I, Ricardi U et al Changes in patterns of practice for prostate cancer radiotherapy in Italy 1995–2003. A survey of the Prostate Cancer Study Group of the Italian Radiation Oncology Society. *Tumori* 2014; 100: 31–37. 10.1700/1430.15812 24675488

[34] Lu W, Olivera GH, Chen Q, Ruchala KJ, Haimerl J, Meeks SL et al Deformable registration of the planning image (kVCT) and the daily images (MVCT) for adaptive radiation therapy. *Phys Med Biol* 2006; 51: 4357–4374. 10.1088/0031-9155/51/17/015 16912386

[35] Zelefsky MJ, Levin EJ, Hunt M, Yamada Y, Shippy AM, Jackson A et al. Incidence of late rectal andurinary toxicities after three-dimensional conformal radiotherapy andintensity-modulated radiotherapy for localized prostate cancer. *Int J Radia Oncol Biol Phys* 2008; 70: 1124–1129. 10.1016/j.ijrobp.2007.11.044 18313526

[36] Dearnaley DP, Sydes MR, Graham JD, Aird EG, Bottomley D, Cowan RA et al Escalated-dose versus standard-dose conformal radiotherapy in prostate cancer: first results from the MRC RT01 randomised controlled trial. Lancet Oncol 2007; 8: 475–487. 10.1016/S1470-2045(07)70143-2 17482880

[37] Kuban DA, Tucker SL, Dong L, Starkschall G, Huang EH, Cheung MR et al Long-term results of the M. D. Anderson randomized dose-escalation trial for prostate cancer. *Int J Radiat Oncol Biol Phys* 2008; 70: 67–74. 10.1016/j.ijrobp.2007.06.054 17765406

[38] Aluwini S, Pos F, Schimmel E, Krol S, van der Toorn PP, de Jager H et al Hypofractionated versus conventionally fractionated radiotherapy for patients with prostate cancer (HYPRO): latetoxicity results from a randomised, non-inferiority, phase 3 trial. Lancet Oncol 2016; 17: 464–474. 10.1016/S1470-2045(15)00567-7 26968359

[39] Dearnaley D, Syndikus I, Mossop H, Khoo V, Birtle A, Bloomfield D et al Conventional versus hypofractionated high-dose intensity-modulated radiotherapy for prostate cancer: 5-year outcomes of the randomised, non-inferiority, phase 3 CHHiP trial. Lancet Oncol 2016; 17: 1047–1060. 10.1016/S1470-2045(16)30102-4 27339115 PMC4961874

[40] Arcangeli G, Saracino B, Arcangeli S, Gomellini S, Petrongari MG, Sanguineti G et al. Moderate Hypofractionation in High-Risk, Organ-Confined Prostate Cancer: Final Results of a Phase III Randomized Trial. *J Clin Oncol* 2017; 35:1891–1897. 10.1200/JCO.2016.70.4189 28355113

[41] De Bari B, Arcangeli S, Ciardo D, Mazzola R, Alongi F, Russi EG et al Extreme hypofractionation for early prostate cancer: Biology meets technology. *Cancer Treat Rev* 2016; 50: 48–60. 10.1016/j.ctrv.2016.08.005 27631875

[42] Fellin G, Mirri MA, Santoro L, Jereczek-Fossa BA, Divan C, Mussari S et al Low dose rate brachytherapy (LDR-BT) as monotherapy for early stage prostate cancer in Italy: practice and outcome analysis in a series of 2237 patients from 11 institutions. Br J Radiol 2016; 89: 20150981 10.1259/bjr.20150981 27384381 PMC5124913

[43] Tagliaferri L, Kovács G, Aristei C, De Sanctis V, Barbera F, Morganti AG et al. Current state of interventional radiotherapy (brachytherapy) education in Italy: results of the INTERACTS survey. *J Contemp Brachytherapy*. 2019; 11: 48–53. 10.5114/jcb.2019.83137 30911310 PMC6431105

