# Supplementary material for: Treatment paths for localised prostate cancer in Italy: The results of a multidisciplinary, observational, prospective study (Pros-IT CNR)
Source: PLoS One. 2019 Nov 1;14(11):e0224151. doi: 10.1371/journal.pone.0224151 (PMC6824566; doi:10.1371/journal.pone.0224151)
Supplement: S1 Table — (DOC) [file pone.0224151.s001.doc]

|  | **a.** Surgery  alone (n=578) | **b.** (Surgery and RT) or  (Surgery, RT and ADT)  (n=65) | **c.** Exclusive RT  (n=345) | **d.** RT and ADT  (n=224) | **e.** ADT alone  (n=110) | **f.** AS  (n=90) | p-value | Significant  post-hoc |
| --- | --- | --- | --- | --- | --- | --- | --- | --- |
| Age at diagnosis, years (SD) | 64.6±6.6 | 64.6±7.2 | 72.9±5.2 | 72.9±5.5 | 74.3±6.1 | 67.2±7.4 | <0.0001 | a vs (c,d,e,f)  b vs (c,d,e,f)  c vs (e,f)  d vs (e,f)  e vs f |
| 3+moderate/severe comorbidities, n (%) | 62 (10.7) | 7 (10.8) | 62 (18.0) | 44 (19.7) | 24 (22.2) | 13 (14.6) | 0.0013 | a vs (c,d,e) |
| Number of drugs, median (Q1,Q3) | 1 (0, 3) | 1 (0, 2) | 2 (1, 4) | 3 (1, 5) | 2 (1, 4) | 1 (0, 3) | <0.0001 | a vs (c,d,e)  b vs (c,d,e)  d vs e |
| T staging at diagnosis, n (%)  T1  T2  T3 or T4 | 334 (60.0)  210 (37.7)  13 (2.3) | 18 (29.0)  28 (45.2)  16 (25.8) | 131 (40.3)  157 (48.3)  37 (11.4) | 57 (26.4)  98 (45.4)  61 (28.2) | 27 (27.8)  48 (49.5)  22 (22.7) | 75 (88.2)  10 (11.8)  0 (0.0) | <0.0001 | a vs (b,c,d,e,f)  b vs f  c vs (d,f)  e vs f |
| Gleason score diagnosis, n (%)  ≤6  3+4  4+3  ≥8 | 291 (50.8)  142 (24.8)  78 (13.6)  62 (10.8) | 18 (28.1)  13 (20.3)  13 (20.3)  20 (31.3) | 156 (45.9)  87 (25.6)  51 (15.0)  46 (13.5) | 38 (17.0)  58 (26.0)  42 (18.8)  85 (38.1) | 19 (17.8)  26 (24.3)  12 (11.2)  50 (46.7) | 81 (93.1)  2 (2.3)  2 (2.3)  2 (2.3) | <0.0001 | a vs (b,d,e,f)  b vs (c,f)  c vs (d,e,f)  e vs f |
| PSA at diagnosis, ng/mL  median (Q1, Q3) | 6.5 (5, 9.1) | 7.5 (5.9, 12) | 7 (5.2, 10) | 9 (6.3, 14.3) | 10.2 (7, 21) | 6.2 (4.8, 7.7) | <0.0001 | a vs (b,d,e)  b vs (e,f)  c vs (d,e,f)  d vs (e,f)  e vs f |
| Intermediate and high risk cases according to D’Amico classification *, n (%) | 386 (68.2) | 55 (85.9) | 268 (79.3) | 215 (97.3) | 97 (92.4) | 17 (20.2) | <0.0001 | a vs (b,c,d,e,f)  b vs (d,f)  c vs (e,f)  d vs f  e vs f |
|  |  |  |  |  |  |  |  |  |
| UCLA PCI UF, mean ± SD | 95.4±13.1 | 84.2±22.8 | 92±16.9 | 92.3±16.9 | 92±15.9 | 94.3±14 | <0.0001 | a vs (b,c,d,e)  b vs (c,d,e,f) |
| UCLA PCI UB, mean ± SD | 92.2±20.4 | 79.7±28.1 | 86.5±24.4 | 84.7±25.9 | 82.9±28.4 | 92.9±16.1 | <0.0001 | a vs (b,c,d,e)  b vs (c,f)  c vs f  d vs f  e vs f |
| UCLA PCI BF, mean ± SD | 95.8±10.7 | 91.8±14.1 | 92±15.1 | 91.6±15.4 | 90.7±15.4 | 95.2±11.6 | <0.0001 | a vs (b,c,d,e)  d vs f  e vs f |
| UCLA PCI BB, mean ± SD | 95.7±14 | 90.4±22 | 93±18.2 | 89.7±23.5 | 88.4±23.4 | 95.5±15.4 | 0.0001 | a vs (b,c,d,e)  d vs f  e vs f |
| UCLA PCI SF, mean ± SD | 61.8±28.3 | 45.3±36 | 38.6±30.3 | 37.9±29.4 | 28.9±28.5 | 60.7±29.8 | <0.0001 | a vs (b,c,d,e)  b vs (e,f)  c vs (e,f)  d vs (e,f)  e vs f |
| UCLA PCI SB, mean ± SD | 67.4±33.6 | 59.9±37.4 | 59.7±36.5 | 57.9±35 | 64.2±36 | 72.7±29.9 | 0.0005 | a vs (c,d)  c vs f  d vs f |
| SF-12 PCS, mean ± SD | 53.3±6.1 | 52.2±6.6 | 50.9±7.8 | 50.5±8.3 | 47.5±9.1 | 52.3±6.7 | 0.0195 | a vs (c,d,e)  b vs e  c vs (e,g)  d vs e  e vs f |
| SF-12 MCS, mean ± SD | 48.7±9.6 | 47.4±9.4 | 50.3±9.5 | 49.3±9.8 | 49.2±9.8 | 50.6±8.9 | <0.0001 | a vs c  b vs (c,f) |
|  |  |  |  |  |  |  |  |  |
| Patient enrolled by, n (%)  Urologist  Radiation Oncologist  Medical Oncologist | 548 (94.8)  28 (4.8)  2 (0.4) | 40 (61.5)  25 (38.5)  0 (0.0) | 29 (8.4)  310 (89.9)  6 (1.7) | 37 (16.5)  181 (80.8)  6 (2.7) | 52 (47.3)  49 (44.6)  9 (8.2) | 52 (57.8)  37 (41.1)  1 (1.1) | <0.0001 | a vs (b,c,d,e,f)  b vs (c,d,e)  c vs (d,e,f)  d vs (e,f) |
| Presence of a Urology Unit, n (%) | 573 (99.1) | 62 (95.4) | 291 (84.4) | 213 (95.1) | 102 (92.7) | 89 (98.9) | <0.0001 | a vs (c,d,e)  d vs (e,f) |
| Presence of a Radiation Oncology Unit, n (%) | 418 (72.3) | 50 (76.9) | 326 (94.5) | 214 (95.5) | 94 (85.5) | 69 (76.7) | <0.0001 | a vs (c,d)  b vs (d,e)  d vs (e,f) |
| Presence of a Medical Oncology Unit, n (%) | 499 (86.3) | 56 (86.2) | 332 (96.2) | 222 (99.1) | 105 (95.5) | 87 (96.7) | <0.0001 | a vs (c,d)  b vs (d,e) |
| Presence of a Prostate Unit, n (%) | 109 (18.9) | 10 (15.4) | 43 (12.5) | 31 (13.8) | 22 (20.0) | 23 (25.6) | 0.0184 | d vs f |

Table 1. Patients’ characteristics stratified according to the treatment pathway

SD: Standard Deviation; BMI: Body Mass Index; Q1: Quartile 1; Q3: Quartile 3; SF-12: Short-Form Health Survey; PCS: Physical Component Subscale; MCS: Mental Component Subscale; UCLA: University of California Los Angeles-Prostate Cancer Index; UF: Urinary Function; UB: Urinary Bother; BF: Bowel Function; BB: Bowel Bother; SF: Sexual Function; SB: Sexual Bother

* Percentage with T staging at diagnosis higher than T2a or Gleason Score at diagnosis >=3+4 or PSA at diagnosis > 10 ng/mL, i.e., intermediate and high risk cases according to D’Amico classification ^44^
